# Two types of interneurons in the mouse lateral geniculate nucleus are characterized by different h-current density

**DOI:** 10.1038/srep24904

**Published:** 2016-04-28

**Authors:** Michael Leist, Maia Datunashvilli, Tatyana Kanyshkova, Mehrnoush Zobeiri, Ania Aissaoui, Manuela Cerina, Maria Novella Romanelli, Hans-Christian Pape, Thomas Budde

**Affiliations:** 1 Institut für Physiologie I, Westfälische Wilhelms-Universität, Robert-Koch-Str. 27a, 48149 Münster, Germany; 2 Laboratory of Sleep-Wakefulness Cycle Studies, Faculty of Arts and Science, Ilia State University, Kakutsa Cholokashvili Ave 3/5, Tbilisi 0162, Georgia; 3 Institut für Physiologie I – Neuropathophysiologie, Albert-Schweitzer Campus 1, 48149 Münster, Germany; 4 Department of Neurosciences, Psychology, Drug Research and Child Health, University of Florence, Via Ugo Schiff 6, 50019 Sesto Fiorentino, Italy

## Abstract

Although hyperpolarization-activated cyclic nucleotide-gated cation (HCN) channels and the corresponding h-current (*I*_h_) have been shown to fundamentally shape the activity pattern in the thalamocortical network, little is known about their function in local circuit GABAergic interneurons (IN) of the dorsal part of the lateral geniculate nucleus (dLGN). By combining electrophysiological, molecular biological, immunohistochemical and cluster analysis, we characterized the properties of *I*_h_ and the expression profile of HCN channels in IN. Passive and active electrophysiological properties of IN differed. Two subclasses of IN were resolved by unsupervised cluster analysis. Small cells were characterized by depolarized resting membrane potentials (RMP), stronger anomalous rectification, higher firing frequency of faster action potentials (APs), appearance of rebound bursting, and higher *I*_h_ current density compared to the large IN. The depolarization exerted by sustained HCN channel activity facilitated neuronal firing. In addition to cyclic nucleotides, *I*_h_ in IN was modulated by PIP_2_ probably based on the abundant expression of the HCN3 isoform. Furthermore, only IN with larger cell diameters expressed neuronal nitric oxide synthase (nNOS). It is discussed that *I*_h_ in IN is modulated by neurotransmitters present in the thalamus and that the specific properties of *I*_h_ in these cells closely reflect their modulatory options.

The dorsal part of the lateral geniculate nucleus (dLGN) represents the main thalamic relay station for visual information in mammals^1^. The vast majority (70–80%) of dLGN neurons consists of thalamocortical (TC) neurons which transmit and process sensory information from the retinal ganglion cells to the primary visual cortex. Depending on species and functional area, thalamic nuclei contain up to 30% local circuit GABAergic interneurons (IN), which inhibit TC neurons^2^. Since IN cell bodies are typically among the smallest of the thalamus and reveal low contrast in differential interference contrast (DIC)-based video imaging, direct targeting in brain slices is laborious^3^,^4^. Indeed, indirect measures like the analysis of IN-mediated IPSPs in thalamocortical relay (TC) neurons and acute isolation techniques allowing visual morphological classification were frequently used to analyze their synaptic function^5^,^6^ and ion channel repertoire^7^,^8^,^9^, respectively. The generation of GAD67-EGFP knock-in mice greatly improved functional analysis of thalamic IN^10^,^11^,^12^,^13^. Nevertheless mathematical models for mouse dLGN IN were based on the assessment of electrophysiological properties of a small number of cells^14^. In particular the role of HCN channels which constitute the molecular basis of the hyperpolarization-activated inward current (*I*_h_), is not well understood. While *I*_h_ has been suggested to contribute to the peculiar bursting pattern of IN, a possible involvement in the generation of intrinsic oscillations has largely been excluded^4^,^14^,^15^. However some of the fundamental functions and properties of *I*_h_, like the contribution to the resting membrane potential, its modulation by intracellular second messengers and the expression profile of different HCN channel subunits in IN has yet not been addressed.

Classically dLGN IN somata were described as small fusiform or oval with primary dendrites emerging from opposite poles in rats^16^, mice^17^ and cats^18^, termed type II/class B cells in rodents and class III cells in cats. Several morphological and immunohistochemical properties found for IN, point to the existence of at least two subclasses of cells. In the cat^19^, ferret^20^ and rat^21^ visual thalamus two types of interneurons were identified based on the expression of neuronal nitric oxide synthase (nNOS). The findings that the dendritic morphology of nNOS-positive IN in cats (termed class V cells) was different from class III cells, that nNOS-positive IN in ferret frequently had multipolar morphology, that nNOS-positive IN in rat and cat had significantly larger somatic areas, and that some glutamate decarboxylase (GAD)-positive cells in rats^8^ revealed a equilateral triangle shape further point to the presence of functionally different classes of IN in dLGN.

By combining electrophysiological recordings, molecular biological analysis, immunohistochemical staining and cluster analysis, we here aimed to characterize the properties of *I*_h_ in relation to possible IN subclasses in mouse dLGN.

## Results

### Classification of GAD67-EGFP-positive neurons in dLGN

**Figure 1:**
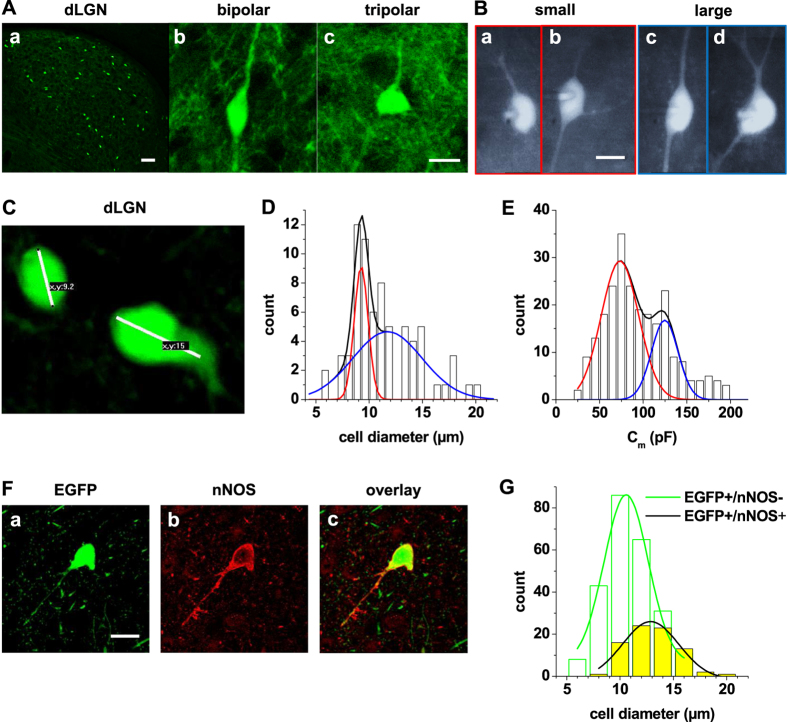
Size distribution and nNOS expression of EGFP-labeled IN in dLGN. (**A**) Coronal section of dLGN from a P21 GAD67-EGFP mouse revealing the scattered distribution of IN (**Aa**; scale bar 100 μm). Higher spatial resolution images revealed spindle-shaped (**Ab**) and tripolar (**Ac**); scale bar 10 μm) neurons. (**B**) Fluorescent images of patched IN showing small (**Ba,Bb**) and large (**Bc,Bd**) cells with both shapes (scale bar 10 μm). (**C**) Confocal image of two EGFP-expressing IN in dLGN with software based determination of cell diameters indicating the presence of small and large cells. (**D**) Size-frequency distribution of soma diameters of EGFP-expressing IN. The histogram was obtained from the two-dimensional analysis of the somatic diameter. The ordinate represents the number of cells within each 0.75 μm diameter class along the abscissa. Note the bimodal size distributions for the total IN population in dLGN. (**E**) Frequency distribution of membrane capacitance of EGFP-expressing IN recorded under current clamp conditions. The ordinate represents the number of cells within each 10 pF class along the abscissa. Note the bimodal membrane capacitance distributions for the total IN population in dLGN. (**F**) Immunofluorescent staining for the presence of nNOS (**Fb**) in EGFP-expressing cells (**Fa**) in dLGN. The overlay (**Fc**) reveals co-expression in a fraction of IN. Scale bar represents 15 μm. (**G**) Size-frequency distribution of soma diameters of cells only expressing EGFP (green bars) and cells double-labeled for EGFP and nNOS (yellow bars). The histogram was obtained from the two-dimensional analysis of the somatic diameter. The ordinate represents the number of cells within each 2 μm diameter class along the abscissa. Solid lines represent Gauss fits to the data points. Please note that nNOS positive IN tend to have larger soma diameters.

In dLGN cell bodies and dendrites of EGFP-labeled neurons were reliably detected by fluorescent microscopy and we refer to them as local GABAergic IN in the following. These cells were scattered throughout the dLGN with a density of 26 ± 1 cells/180 ± 12 μm^2^ (mean ± standard deviation; n = 9 coronal sections, 30 μm thickness; Fig. 1Aa). Fluorescent microscopic inspection revealed cells with the classical morphology of rodent interneurons (spindle-shaped soma with primary dendrites originating from opposite poles; Fig. 1Ab,Ba,Bc). In addition, cells with a tripolar appearance were found (triangular soma with three primary dendrites; Fig. 1Ac,Bb,Bd). Confocal imaging and measurement of the length of the cell body (Fig. 1C), revealed a size distribution of the somatic diameter which was best fit by a bimodal Gaussian function (μ_1_ = 9.3 μm, σ_1_ = 1.3 μm; μ_2_ = 11.7 μm, σ_2_ = 6.5 μm) thereby revealing two populations of IN (Fig. 1D). In addition C_m_ values (as an indirect measure of cell size) of IN determined by patch-clamp recordings revealed a bimodal distribution (μ_1_ = 73.4 pF, σ_1_ = 42.9 pF; μ_2_ = 124.7 pF, σ_2_ = 30.3 pF; Fig. 1E), thereby further supporting the existence of two IN populations based on cell size. To identify an additional independent parameter for IN classification, we stained thalamic slices for the presence of nNOS (Fig. 1F). A subset of IN (about 20%; green fluorescence, Fig. 1Fa) was found to express nNOS in somatic and dendritic areas (Fig. 1Fb). The mean cell diameter of EGFP labeled cell positively stained for nNOS (13.1 ± 0.2 μm, n = 80) was significantly (P < 0.001) larger compared to nNOS negative cells (10.8 ± 0.1 μm, n = 244; Fig. 1G).

**Figure 2:**
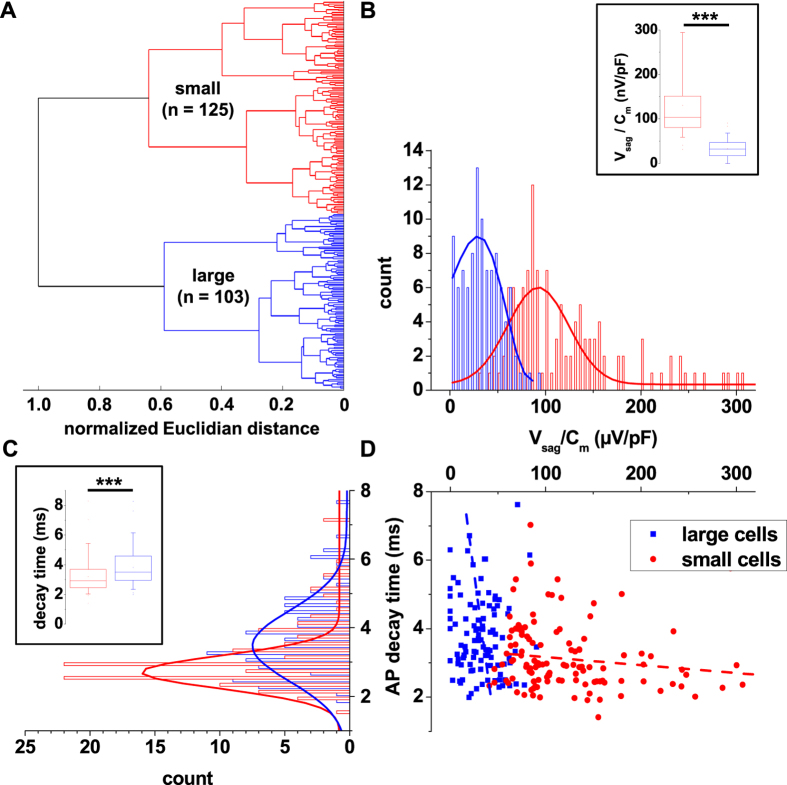
Cluster analysis classification of EGFP-expressing IN in dLGN. (**A**) Ward’s unsupervised analysis was used with physiological parameters from Tab. S1 to classify 228 EGFP-labeled IN. The cut-off value at the normalized Eulclidian distance of 0.7 (i.e., 70% the maximal Euclidian distance) separated the dendrogram into small and large neurons. (**B**) The frequency distribution of the amplitude of the V_sag_ amplitude normalized to cell size (voltage sag amplitude divided by membrane capacitance) of EGFP-expressing IN after K-means correction revealed two populations of cells (bin size 5 μV/pF). Solid lines represent Gauss functions fitted to the data points. The inset shows the box plot representation of this parameter (t-test, two tailed: p < 0.001). (**C**) The frequency distribution of the decay time of the first AP induced by a positive current step (+80 pA) of GFP-expressing IN after K-means correction revealed two populations of cells (bin size 0.2 ms). Solid lines represent Gauss functions fitted to the data points. The inset shows the box plot representation of this parameter (t-test, two tailed: p < 0.001). (**D**) Scatter plot depicting the normalized voltage sag vs. AP decay time for each IN included for cluster analysis after K-means correction. Two cell populations are clearly visible.

Since the findings described above point to the presence of two classes of IN in mouse dLGN, we next used passive and active membrane properties (Supplementary Table S1) to perform an unsupervised cluster analysis (Fig. 2A). Based on 12 functional parameters we applied Ward’s method which has been used in the classification of interneurons before^22^,^23^. Using a cut-off value of 0.7 (i.e., 70% of the maximal Euclidian distance), the resulting hierarchical tree diagram clearly separated two groups of cells. The groups were designated as small and large IN and represented 125 and 103 cells, respectively. Ward’s method required no assumptions with respect to the number of clusters into which observations should be classified. However, miss-assigned cells were not corrected by this method due to its iterative nature. Therefore, the K-means algorithm was used to eliminate potential errors of the Ward´s clustering process^23^,^24^,^25^. Following correction 15 cells of each group were reassigned to the other cluster (Table 1). In order to determine whether the assignment of IN to two groups was the optimal outcome of the clustering process, trails for up to 5 groups of IN were analyzed for comparison. After K-means correction, the mean silhouette value for two clusters was 0.3205, while lower values (indicating loss of clustering quality) were achieved for three (0.2545), four (0.2054) or five (0.2288) groups of IN. Therefore the subdivision of IN into two groups was retained in the following. The segregation between the two groups of neurons was best demonstrated by scatter plotting V_sag_ normalized to C_m_ vs. the AP decay time yielding two clearly separated clusters (Fig. 2B–D). When cell morphology was mapped on the two clusters, bipolar and tripolar cells were found in both populations. These findings indicate that mouse dLGN IN are separated in two clusters mainly based on cell size and the degree of anomalous rectification. The basic electrophysiological properties of these two groups were analyzed in the following.

### Electrophysiological properties of small and large IN

**Figure 3:**
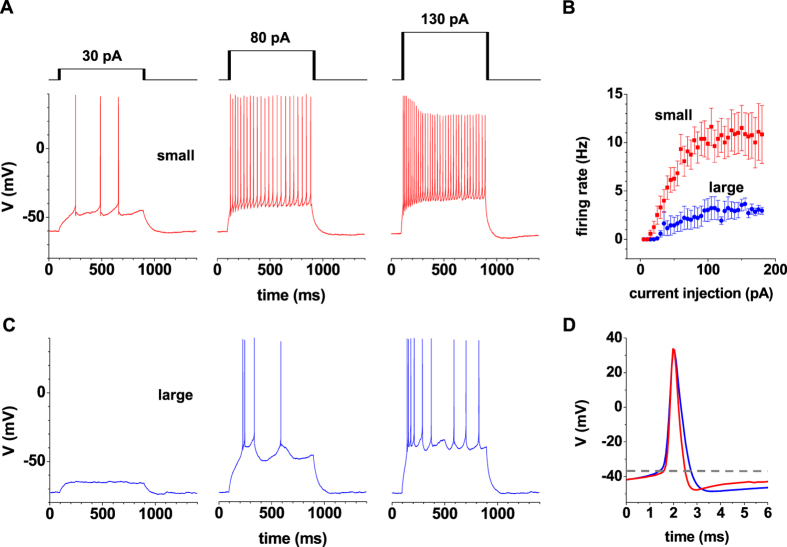
Functional properties of EGFP-expressing IN in dLGN. (**A,C**) Examples of voltage responses to increasing current injections (as indicated) in a small (**A**) and a large (**C**) IN are shown. Cells were kept at RMP. (**B**) Both groups of IN revealed a saturating relationship between spike frequency and current injection. (**D**) Details of the first AP evoked by a depolarizing current injection (+80 pA) are shown in an overlap at high temporal resolution. The mean AP shape form in small (n = 15, red line) IN is typically shorter in comparison to large IN (n = 15, blue line).

**Figure 4:**
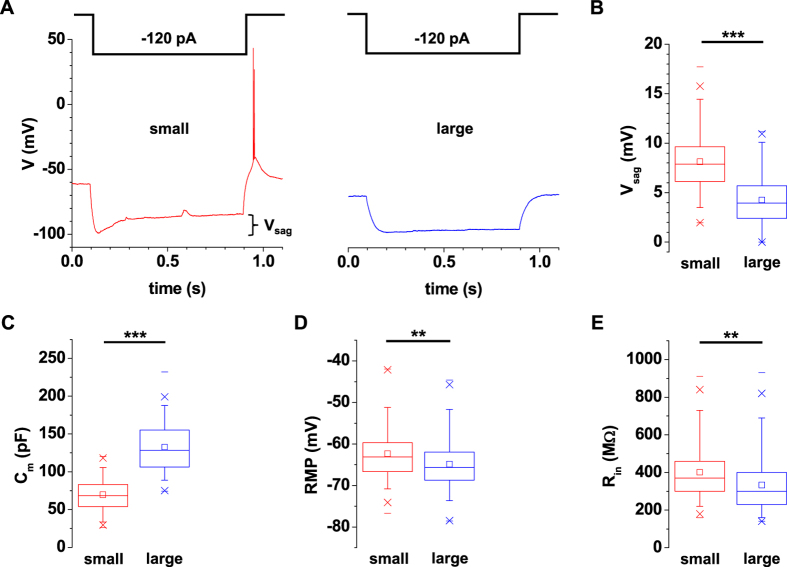
Passive membrane properties of EGFP-expressing IN in dLGN. (**A**) Voltage responses to negative current pluses (hyperpolarizing the membrane potential close to −100 mV) revealed anomalous rectification in both types of IN. Small IN typically revealed a rebound low threshold Ca^2+^ spike (LTS). (**B–E**) Several membrane parameters including voltage sag (V_sag_, (**B**) t-test, two tailed: P < 0.001) membrane capacitance (C_m_, (**C**) t-test, two tailed: p < 0.001) resting membrane potential (RMP, **D**; t-test, two tailed: P < 0.01) and membrane input resistance (R_in_, (**E**) t-test, two tailed: P < 0.01) revealed significant differences between small (red box plots) and large (blue box plots) IN.

Neuronal firing pattern were analyzed by performing whole cell patch-clamp recordings from IN in current-clamp mode (Figs 3A,C and 4A). Depolarizing square-wave current pulses of increasing amplitudes evoked trains of APs which revealed a linear rise in AP frequency for current injections up to about 100 pA (Fig. 3B) as has been reported for IN in different species before^17^,^26^,^27^. Thereafter IN reached maximum discharge rates. Typically large IN fired less APs. Some IN revealed oscillatory bursting in response to depolarizing current pulses (Fig. 3C, middle panel) which has been described as the prevailing firing pattern in rat IN^4^. In addition more complex firing patterns were observed in some IN including stuttering fast spiking, and adapting spiking^28^. Analysis of the first AP elicited by a + 80 pA depolarizing pulse revealed shorter duration in small IN (Fig. 2D). The decay time measured from the peak of the AP to the level of the fast afterhyperpolarization (fAHP) was significantly different (small IN: 3.2 ± 0.1 ms, n = 125; large IN: 3.8 ± 0.1 ms, n = 103; P < 0.001, Figs 2C and 3D). Furthermore, small but not large IN revealed moderate rebound bursting^29^ upon completion of a hyperpolarizing pulse (Fig. 4A). A clear V_sag_ (representing activity of *I*_h_) with a mean amplitude of 8.1 ± 0.3 mV (n = 125) was seen in small IN, while large IN revealed significantly smaller V_sag_ amplitudes (4.2 ± 0.3 mV, n = 103; P < 0.001, Fig. 4A,B).

Passive membrane properties were assessed in the following. Two populations of C_m_ values (representing cell size) were in good agreement with small (69.5 ± 1.9 pF, n = 125) and large (132.7 ± 3.2 pF, n = 103) IN (Fig. 4C). The mean RMP of small IN (−62.4 ± 0.6 mV, n = 125) was significantly more depolarized than values for large IN (−64.8 ± 0.7 mV, n = 103; P < 0.01; Fig. 4D). R_in_ of small IN (401.3 ± 13.8 MΩ, n = 125) was about 1.2 times higher (P < 0.01) compared to large cells (333.1 ± 15.4 MΩ, n = 103; Fig. 4E).

These data point to distinct differences in membrane characteristics in two types of IN which seem to be based at least partially on differences in *I*_h_ properties.

### Basic voltage-dependent properties of *I*_h_ in IN

**Figure 5:**
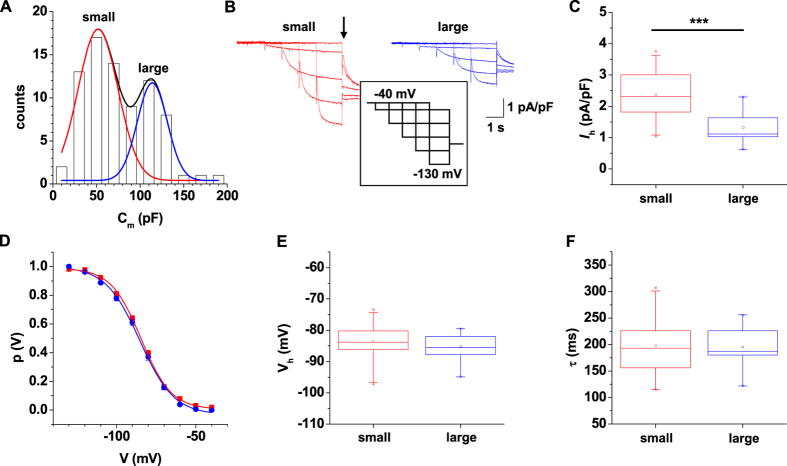
Properties of *I*_h_ in EGFP-expressing IN in dLGN. (**A**) Frequency distribution of membrane capacitance of EGFP-expressing IN recorded under voltage clamp conditions. The ordinate represents the number of cells within each 20 pF class along the abscissa. Note the bimodal membrane capacitance distribution for the total IN population in dLGN. (**B**) Representative *I*_h_ recordings from IN in dLGN from small (red traces) and large (blue traces) IN are shown. (**C**) Mean *I*_h_ current density from small and large IN determined at −130 mV (Mann-Whitney test, two tailed: P < 0.001). (**D**) Mean steady-state activation curves from small (red data points) and large (blue data points) IN are shown. Solid lines represent best approximations of Boltzmann functions to the data points. (**E,F**) Box plots of the potentials of half-maximal activation (**E**) Mann-Whitney test, two tailed: P = 0.38) and time constants of activation (**F**; Mann-Whitney test, two tailed: P = 0.98) revealed no significant differences between small (red symbols) and large (blue symbols) IN.

The differences in V_sag_ amplitudes in large and small IN pointed to possible alterations in *I*_h_ properties. Therefore we recorded *I*_h_ under voltage-clamp conditions in the presence of 0.5 mM Ba^2+^ in order to block two-pore domain and inward rectifier K^+^ channels^30^,^31^. Under these conditions the parameter distribution of C_m_ was fitted by a bimodal Gaussian function (μ_1_ = 52 pF, σ_1_ = 46 pF; μ_2_ = 114 pF, σ_2_ = 34 pF; n = 78; Fig. 5A). Therefore cells with C_m_ < 90 pF and C_m_ > 90 pF were regarded as small and large cells, respectively. To determine the properties of *I*_h_ in IN, cells were held at a potential of −40 mV and challenged by hyperpolarizing voltage steps of increasing (ΔV = −10 mV) amplitude and decreasing (Δt = −0.5 s) duration (5.5 s at −50 mV to 1.5 s at −130 mV) followed by a constant step to −100 mV (Fig. 5B, inset). Analyses of activating and deactivating currents revealed significant differences between the two IN subtypes for current density (small cells: *I*_h_ = 2.4 ± 0.1 pA/pF, n = 33; large cells: *I*_h_ = 1.3 ± 0.1 pA/pF, n = 14, p < 0.001; Fig. 5C) but not for the voltage-dependency of activation (small cells: V_h_ = −83.5 ± 1.0 mV, n = 33; large cells: V_h_ = −85.2 ± 1.3 mV, n = 14; Fig. 5D,E) and activation kinetics (small cells: τ = 198 ± 11 ms, n = 33; large cells: τ = 195 ± 12 ms, n = 14; Fig. 5F). These data indicated that *I*_h_ current density is different between the two IN subtypes.

The reversal potential was determined by measuring the fully activated *I-V* relationship of the deactivating current at different membrane potentials (supplementary Figure S1A, S1B). Since this current largely consisted of *I*_h_, the intersection with zero line indicated the potential where the driving force for current through HCN channels (E_h_) is zero^32^. Under the present experimental conditions E_h_ was not significantly different between small (−32.6 ± 1.8 mV; n = 15) and large (−29.2 ± 0.6 mV; n = 5) IN.

These data indicate that IN generate a small-sized and fast *I*_h_ and that a reduced *I*_h_ current density is the basis for weak anomalous rectification in large IN.

### Modulation of *I*_h_ by intracellular cyclic nucleotides, phospholipids and channel blockers

**Figure 6:**
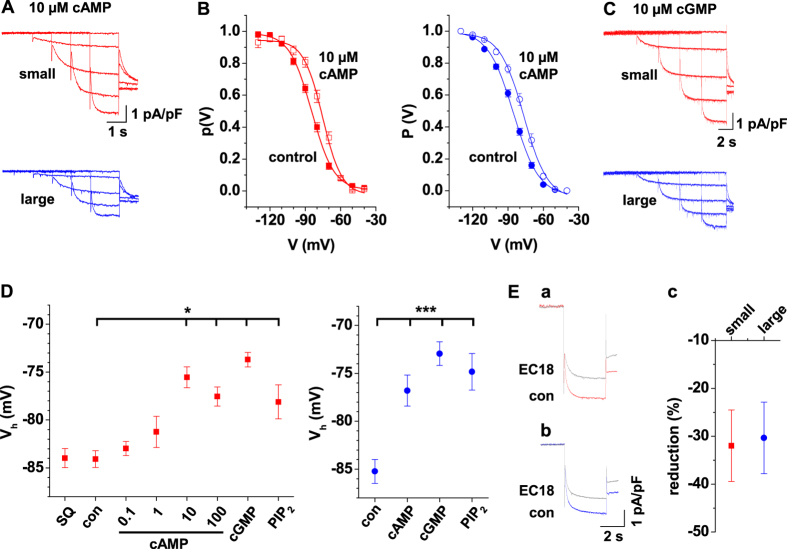
Modulation and block of *I*_h_ in IN in dLGN. (**A,C**) Representative current traces from small (red traces) and large (blue traces) IN in the presence of 10 μM intracellular cAMP (**A**) or cGMP (**C**). (**B**) Mean steady-state activation curves from small (red symbols) and large (blue symbols) IN under control conditions (closed symbols) and with 10 μM cAMP (open symbols) added to the pipette solution. (**D**) V_h_ values of small (red symbols) and large (blue symbols) under control conditions (con), at different cAMP concentrations (included to the pipette solution), preincubation of slices with the adenylyl cyclase inhibitor SQ22536 (SQ) and with cGMP or PIP_2_ added to the pipette solution determined from independent populations of IN (as indicated; one way ANOVA for small IN: F = 10.86; P < 0.0001; Tukey’s post hoc: Con vs. cAMP 10 μM, P < 0.001; con vs. cAMP 100 μM, P < 0.01; con vs. cGMP, p < 0.001; con vs PIP_2_, P < 0.05; one way ANOVA for large IN: F = 14.24; P < 0.0001; Bonferroni’s post hoc: con vs. cAMP, P < 0.001; con vs. cGMP, P < 0.001; con vs. PIP_2_, P < 0.001). (**E**) Current traces evoked by voltage steps to −130 mV in the presence (grey trace) and absence (small IN: red trace, **Ea**) large IN: blue trace, (**Eb**) of the *I*_h_ blocker EC18 (30 μM). Mean value of *I*_h_ reduction by extracellular application of EC18 (**Ec**) Mann-Whitney test, two tailed: p = 0.89).

Modulation by intracellular cyclic nucleotides is a hallmark of *I*_h_ and HCN channels in different preparations and seems to reflect the relative contribution of the different HCN isoforms to native currents^33^. To examine the effect of cAMP in small IN, *I*_h_ was recorded (Fig. 6A, upper panel) while the pipette solution contained defined concentrations (0.1, 1, 10, or 100 μM; one concentration was tested in an individual cell) of 8-bromo-cAMP^34^,^35^. In comparison to cells recorded under control conditions, activation curves shifted to more depolarized potentials (Fig. 6B, left panel) and V_h_ was significantly more depolarized while the pipette solution contained 10 μM (V_h_ = −75.5 ± 1.1 mV, n = 13) and 100 μM (V_h_ = −77.5 ± 1.0 mV, n = 10) 8-bromo-cAMP, thereby indicating a significant cAMP-sensitivity (Fig. 6D, left panel). For comparison 10 μM 8-bromo-cGMP was added to the pipette solution (Fig. 6C, upper panel) and also resulted in a positive shift of the activation curve (V_h_ = −73.7 ± 0.8 mV, n = 10; Fig. 6D, left panel). Preincubation of slices (2 h) with the adenylyl cyclase inhibitor SQ22536 had no significant effect on *I*_h_ activation (V_h_ = −84.0 ± 1.0 mV, n = 5; Fig. 6D, left panel). Furthermore intracellular application of the phospholipid PIP_2_ (10 μM) via the electrode solution shifted V_h_ to significantly (P < 0.05) more depolarized potentials (−78.1 ± 1.8 mV, n = 7; Fig. 6D, left panel). Next, large IN were recorded with cyclic nucleotides added to the pipette solution (Fig. 6A,C, lower panels). In comparison to cells recorded under control conditions, activation curves shifted to more depolarized potentials (Fig. 6B, right panel) and V_h_ was significantly (p < 0.001) more depolarized (Fig. 6D, right panel) while the pipette solution contained 10 μM 8-bromo-cAMP (V_h_ = −76.8 ± 1.6 mV, n = 10) and 10 μM 8-bromo-cGMP (V_h_ = −72.9 ± 1.2 mV, n = 7), thereby indicating a cyclic nucleotide-sensitivity which was not significantly different from small IN. Addition of PIP_2_ to the recording pipette also resulted in a positive shift of the activation curve (V_h_ = −74.8 ± 1.9 mV, n = 9). Again no differences were found between small and large IN.

In order to assess the composition of *I*_h_ in IN, we used the HCN4-specific channel blocker EC18^36^. Application of EC18 (30 μM) reduced *I*_h_ by = −32.0 ± 7.5% (n = 7) and −30.3 ± 7.5% (n = 4; Fig. 6Ec) in small (Fig. 6Ea) and large (Fig. 6Eb) IN, respectively.

The findings indicate that *I*_h_ of IN is modulated by intracellular cyclic nucleotides and PIP_2_ while inhibition of adenylyl cyclase activity had no effect. HCN4 channels contribute to the current in both groups of IN.

### Influence of *I*_h_ on electrophysiological properties of IN

**Figure 7:**
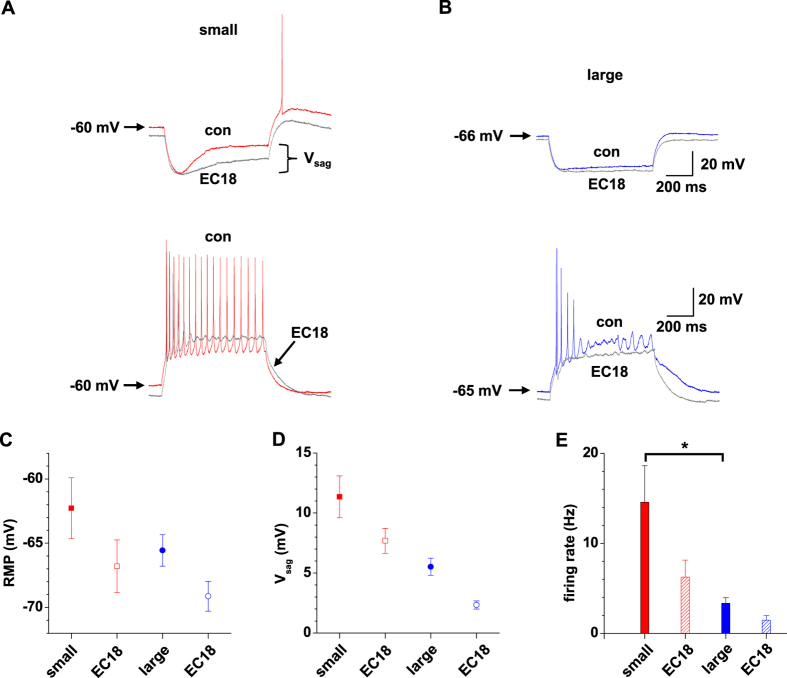
Alterations of passive membrane properties and AP firing due to block of *I*_h_. (**A,B**) Responses to hyperpolarizing (−120 pA; upper panels) and depolarizing (+80 pA; lower panels) current pulses (800 ms duration) in small (**A**) and large (**B**) IN in the presence (grey traces) and absence (small IN: red trace; large IN: blue trace) of the *I*_h_ blocker EC18 (30 μM) are shown. (**C–E**) Mean values of RMP (**C**; the effect of cell size and pharmacology was tested with factorial ANOVA: F for size = 2.49,n.s; F for pharmacology = 5.16, P < 0.05; Bonferroni’s post hoc: n.s.), V_sag_ (**D**; the effect of cell size and pharmacology was tested with factorial ANOVA: F for size = 26.35, p < 0.001; F for pharmacology = 9.88, p < 0.01; Bonferroni’s post hoc: n.s.) and the firing rate during a depolarizing current pulse (+80 pA) from RMP (**E**; the effect of cell size and pharmacology was tested with factorial ANOVA: F for size = 12.49, p < 0.01; F for pharmacology = 5.06, p < 0.05; Bonferroni’s posthoc: small vs. large, p < 0.05) in small and large IN the presence of absence of the *I*_h_ blocker (as indicated).

To assess the functional role of *I*_h_ in IN, we used the general HCN channel blocker ZD7288 and the HCN4-specific blocker EC18^36^ in small IN under current clamp conditions (Fig. 7A). Application of ZD7288 (ΔV = −5.3 ± 1.0 mV, n = 5) and EC18 (ΔV = −4.5 ± 0.7 mV, n = 6) resulted in a hyperpolarization of the RMP (control: −62.3 ± 1.3 mV, n = 11; Fig. 7A,C). Under control conditions a hyperpolarizing current step induced a substantial voltage sag (V_sag_ = 10.4 ± 1.0 mV, n = 11) which was reduced by application of ZD7288 (V_sag_ = 1.3 ± 0.3 mV, n = 5) and EC18 (V_sag_ = 7.7 ± 1.0 mV, n = 6; Fig. 7A,D). The hyperpolarized RMP was associated with a significant (P < 0.05) decrease in the firing rate of APs induced by a depolarizing current step (+80 pA; Fig. 7A,E). For large IN which revealed a more negative RMP (control: −65.6 ± 1.2 mV, n = 6) application of EC18 induced a further membrane hyperpolarization (ΔV = −3.6 ± 0.3 mV, n = 6) which was associated with a reduction in amplitude of the V_sag_ and AP firing (Fig. 7B–E).

These findings indicate that *I*_h_ in IN generates a prominent V_sag_ and tonically depolarizes the membrane potential thereby facilitating AP firing.

### *I*_h_ in TC neurons is different

For comparison *I*_h_ was recorded in TC neurons from GAD67-EGFP mice (supplementary Figure S2B). C_m_ values in TC neurons were 94 ± 3 pF (n = 43; supplementary Figure S2A). The mean steady-state activation curve was similar to that found in IN (supplementary Figure S2C, S2D). However, *I*_h_ in TC neurons revealed much higher current density (supplementary Figure S2E) and slower activation kinetics (supplementary Figure S2F), compared to IN (Fig. 5C,F). Furthermore intracellular application of 10 μM 8-bromo-cAMP (V_h_ = −68.9 ± 1.2 mV, n = 11) and 10 μM 8-bromo-cGMP (V_h_ = −76.5 ± 1.3 mV, n = 12) significantly (p < 0.001) shifted the activation curve to more depolarized potentials while infusion of PIP_2_ had no effect (V_h_ = −83.8 ± 1.9 mV, n = 5; supplementary Figure S2D).

These findings indicate that TC neurons generate a slow and large-sized *I*_h_ which is strongly modulated by cyclic nucleotides but not PIP_2_ (supplementary Figure S2D).

### Expression of HCN channel in IN on mRNA and protein level

**Figure 8:**
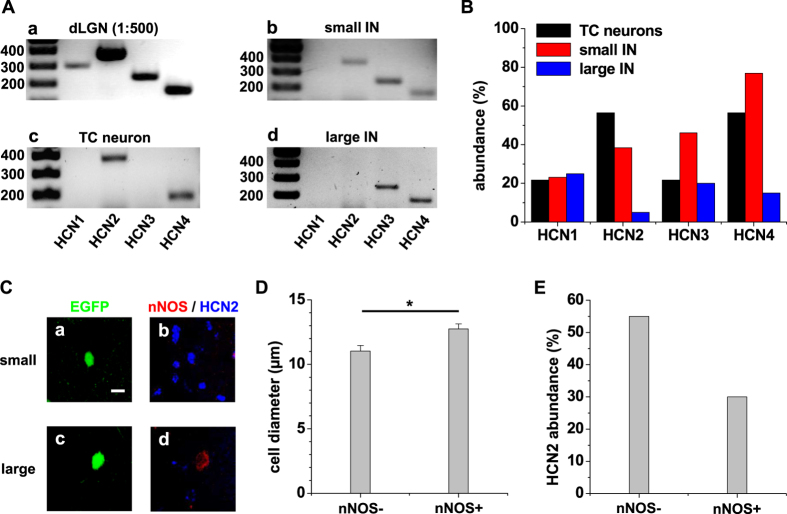
Analysis of HCN channel mRNA and protein expression in IN and TC neurons from dLGN. (**A**) Agarose gels of dLGN tissue ((**Aa)** 1:500 diluted), a pool of 10 TC neurons (**Ac**), a pool of 10 small IN (**Ab**) and a pool of 10 large IN (**Ad**) are shown. A 100 bp ladder starting from 1000 bp was used to indicate band size. (**B**) Quantification of HCN isoforms detected in TC neurons (black bars), small IN (red bars) and large IN (blue bars). (**C**) Confocal images of EGFP-expressing neurons (**Ca,Cc**) stained for the protein expression of nNOS (red fluorescence) and HCN2 (blue fluorescence; (**Cb**,**Cd**) are composites of red and blue fluorescence). Note the lack of nNOS or HCN2 in a small (**Ca,Cb**) and a large (**Cc,Cd**) IN, respectively. The scale bar represents 10 μm. (**D**) Mean values of the somatic diameter of nNOS-negative (nNOS−) and nNOS-positive (nNOS+) cells (as indicated; Mann-Whitney test, two tailed: p < 0.05). (**E**) Quantification of HCN2 detection in nNOS-negative (nNOS−) and nNOS-positive (nNOS+) cells (as indicated).

To determine the molecular basis of *I*_h_, pools of 10 identified small (26 pools; cell diameter <12 μm; Fig. 8Ab) and large (12 pools; cell diameter >12 μm; Fig. 8Ad; cf. Fig. 1D) IN as well as TC neurons (23 pools; Fig. 8Ac) were harvested following acute isolation and subjected to a conventional PCR analysis using HCN1-4-specific primers (Fig. 8Aa). Pools with a positive PCR signal for GAPDH were used for further analysis. In TC neuron pools HCN4 and HCN2 were frequently detectable while HCN3 and HCN1 were less expressed (Fig. 8B, black bars). Small IN were characterized by frequent detection of HCN4 and HCN3 followed by HCN2 and HCN1 (Fig. 8B, red bars). For large IN the abundance for HCN2-4 mRNAs was found to be generally lower compared to small IN (Fig. 8B blue bars). Especially HCN2 was barely detectable. To further characterize HCN channel expression in the two groups of IN (Fig. 8Ca,Cc), we performed immunohistochemical double staining for the presence of nNOS (red fluorescence) and HCN2 (blue fluorescence; Fig. 8Cb,Cd). While nNOS-negative IN (11.0 ± 0.4 μm, n = 42) were significantly (p < 0.05) smaller compared to nNOS-positive IN (12.8 ± 0.4 μm, n = 20; Fig. 8D), HCN2 expression was more frequent in small compared to large IN (Fig. 8E).

These findings indicate that differences in the expression profile of HCN channel isoforms in IN and TC neurons may be the molecular basis for differences in *I*_h_ properties. The low abundance of HCN channel mRNAs in large IN is in accordance with their low *I*_h_ current density.

## Discussion

The findings presented here indicate that dLGN IN are heterogeneous with respect to cell morphology (bipolar vs. tripolar), cell size (small vs. large), the degree of anomalous rectification and rebound bursting (weak vs. strong) as well as current density of *I*_h_ (low vs. high). Indeed, cluster analysis revealed two cell populations with membrane capacitance being the most important segregating parameter. We found that small IN were characterized by a more depolarized RMP, strong anomalous rectification, higher firing frequency of faster AP, rebound bursting, and higher *I*_h_ current density compared to large IN. These findings point to the presence of two IN populations in mice. Although possible functional differences between the groups were not determined yet, it has been shown that the RMP affects the nature of active responses that propagate throughout IN axons and dendrites^37^. Indeed, slightly depolarized IN tended to fire single short-latency spikes, whereas more hyperpolarized IN fire Ca^2+^ spikes which were often associated with long-latency sodium spikes^37^. It is interesting to note that during pharmacological block of glutamate receptors only a fraction of IN can still generate robust Ca^2+^ -dependent plateau potentials thereby further pointing to differential voltage-dependent processes in this cell type^38^.

Here we discovered that an important functional impact of HCN channels is that tonic *I*_h_ activity depolarizes IN thereby reducing the voltage difference to AP threshold and allowing the generation of more AP in response to small depolarizing inputs. The shift of *I*_h_ activation curves by intracellular application of cAMP, cGMP or PIP_2_, points to the possibility of HCN channel modulation by neurotransmitters.

The finding of broader AP with longer lasting fAHP in large IN may also contribute to the lower firing rate in this IN subtype. Mathematical modeling indicates that a reduction in fAHP amplitude is sufficient to increase the firing rate of IN^14^. Since the characteristics of different types of AHPs depend on the interaction between Ca^2+^ and Ca^2+^ -dependent K^+^ channels^39^, both may be different in the two IN clusters.

The finding of different dLGN IN subtypes based on soma size, cell morphology and expression of nNOS fits to the general framework which has been established in the literature, although distinct species-specific differences have been found. Based on Golgi impregnations counterstained for lipofuscin pigmentation as well as GAD and GABA staining, two types of local circuit neurons have been distinguished early on in human^40^,^41^ and cat dLGN^42^,^43^. In addition, nNOS has been detected in a subset of IN in different species^19^,^20^,^21^. It was noted in human dLGN that IN cell bodies were usually spindle-shaped but frequently generated additional root-like protrusions, partially resulting in triangular shapes. In cat dLGN the population of IN was composed of a large proportion of small and a smaller proportion of medium sized neurons. Furthermore the two classes of interneurons in cats differentially react to immunostaining for nNOS^19^. Interestingly, nNOS-positive cells revealed significantly larger soma areas compared to the overall population of IN and appeared to be involved primarily in extraglomerular synapses, whereas nNOS-negative cells may exert their effects primarily within synaptic glomeruli^19^,^21^. With respect to electrophysiological properties it was shown that differences in oscillatory firing found in rat IN were related to significant differences in R_in_, thereby pointing to the possible existence of cell populations with low and high R_in_^4^. Here we extent these findings to mice where two groups of IN were defined by cell size, nNOS expression and a number of passive membrane properties, including R_in_. Furthermore the higher *I*_h_ current density in small IN is in line with strong anomalous rectification, rebound bursting and a depolarized RMP, thereby proving mathematical models^14^. That two groups of IN in mouse dLGN have not been described before may be related to the use of differently generated transgenic mouse lines^12^,^17^, a possible general bias towards the analysis of classical spindle shaped cells and the fact that major markers, like nNOS, exhibit developmental changes in expression^20^. Due to the lack of reliable post hoc identification of nNOS expression in our recorded cells, it is unclear whether the nNOS-positive neurons show the same intrinsic properties as the large IN. The findings that large IN revealed low abundance for HCN2 expression on mRNA level and that only a minority of IN positive for nNOS protein expression was also positive for HCN2 protein expression potentially indicate that nNOS-expressing IN belong to the functional group of large IN. Future studies have to clarify whether nNOS is a clear distinguishing characteristic of the functional subtypes.

In mice dLGN IN belong to a specialized lineage of neurons which are born during midterm of embryogenesis from a prethalamic progenitor domain, reside in the ventral LGN (vLGN) until birth and progressively incorporate into retinogeniculate circuits during the first postnatal week in an activity-dependent manner^13^. Based on molecular, morphological and electrophysiological properties it has been suggested that rodent dLGN IN represent a largely homogeneous cell population^13^,^26^. Beside these unifying aspects, several factors may contribute to diverging findings in different species. While a linear relationship between firing rate and current injection with non-adapting fast tonic firing was described in IN from cat^27^ and mice^17^, no tonic firing but oscillatory activity was seen in most IN in rats^4^. In the present study a linear input vs. firing rate was found for small current injections in addition to a number of complex firing patterns. This heterogeneity seems to be partially based on different recording techniques (intracellular sharp electrodes vs. patch clamp). However, other factors may contribute, including complex branching pattern of IN in mice^17^ thereby influencing the electrical compactness, the degree of intracellular Ca^2+^ buffering used for patch clamping, and the availability of *I*_h_. An increased *I*_h_ conductance is associated with facilitation of repetitive AP generation and rebound bursting^14^. High Ca^2+^ buffering conditions are associated with a decrease in the number of fired AP but an increase in the complexity of observable firing pattern^44^. The latter is in agreement with the multiple firing patterns found in the present study. Furthermore, mathematical modeling suggested that relative differences in conductance values of ion channels, rather than differences in ion channel composition account for distinctions in IN firing properties. This is in line with the present findings and indicates that IN have rather homogenous gene expression profiles^13^.

A strong *I*_h_ is a hallmark of thalamic TC neurons in different species and nuclei with well-defined roles in setting the RMP and generating rhythmic activity pattern as well as identified channel modulators^33^,^34^,^35^,^45^,^46^. In dLGN IN the current was yet less thoroughly characterized with some indication for differences between species^14^,^15^. Originally rat IN were found to express a depolarized *I*_h_ (V_h_ = −80.8 mV) with rather slow activation kinetics (estimated τ ≈ 250 ms at −130 mV)^15^. Based on a small number of recorded cells the corresponding values were V_h_ = −96 mV and τ ≈ 50 ms at −130 mV in mice^14^. Here we found V_h_ = −83.5 mV and τ ≈ 198 ms at −130 mV for small IN, indicating that *I*_h_ in different rodent IN is more similar then formally suggested.

There are striking differences between *I*_h_ in IN and TC neurons, with the current in IN being smaller and faster. With respect to cAMP, modulation of *I*_h_ is less pronounced in IN compared to TC neurons. Basal cAMP levels are not effecting the current in IN but strongly control *I*_h_ in TC neurons^35^. In general the gating of HCN channels by cGMP is not as thoroughly characterized as cAMP but seems to be less effective on HCN channels in expression systems^47^. The different sensitivity between *I*_h_ in IN and TC neurons may be explained by differences in the HCN channel expression profile. While rodent TC neurons are dominated by strongly cAMP-modulated HCN2 and HCN4 channels^48^,^49^, IN revealed at strong mRNA expression of the cAMP-inhibited HCN3 isoform^50^ and the thalamic isoform HCN4^51^, thereby potentially explaining the reduced cAMP-sensitivity of IN. The functional expression of HCN4 channels is confirmed by the blocking effect of the specific HCN4 blocker EC18^36^. Furthermore *I*_h_ in IN, but not TC neurons is positively modulated by PIP_2_, thereby opening up an additional avenue for HCN channel modulation in these cells. It is interesting to note that strong expression of HCN3 channels is a feature which IN share with another GABAergic thalamic cell type, namely the neurons of the intergeniculate leaflet^52^. *I*_h_ in intergeniculate leaflet cells is strongly modulated by PIP_2_ but insensitive to cAMP. It should be noted that the characteristics of *I*_h_ in dLGN TC neurons found in the present study and in wild type littermates (HCN2^+/+^) of HCN2-deficient mice are very similar with respect to steady state activation (V_h_ = −87.6 ± 0.8 mV, n = 17 in HCN2^+/+^; V_h_ = −86.3 ± 0.5 mV, n = 15 in GAD67-EGFP), current density (5.5 ± 0.5 pA/pF, n = 12 in HCN2^+/+^; 6.5 ± 0.4 pA/pF, n = 15 in GAD67-EGFP) and activation kinetics at −130 mV (τ = 413 ± 19 ms, n = 17 in HCN2^+/+^; τ = 539 ± 27 ms, n = 15 in GAD67-EGFP), thereby corroborating the distinction between *I*_h_ in TC neurons and IN^48^.

*I*_h_ exerts a strong depolarizing influence on the RMP of IN. The block of *I*_h_ or endogenous low current density, as in large IN, is accompanied by membrane hyperpolarization and a reduction in AP firing, thereby critically influencing neuronal excitability. The molecular mechanisms of oscillatory activity in IN and TC neurons appear to be quite different. TC neurons generate oscillatory bursting in the delta frequency range at hyperpolarized membrane potentials due to the cyclic interaction of *I*_h_ and the T-type Ca^2+^ current (I_T_)^35^,^53^. IN show oscillatory burst activity at more depolarized membrane potentials with no need of *I*_h_ for direct cyclic interactions^4^. Nevertheless the sustained depolarization generated by HCN channel may augment the depolarized state needed for the occurrence of oscillatory bursting in these cells.

The possible modulation of *I*_h_ in IN is expected to have pronounced effects on the fundamental properties of the cells^15^, but has not been assessed before. Our data demonstrate a dose-dependent shift of *I*_h_ activation curves to more depolarized potentials by cyclic nucleotides and PIP_2_. Therefore neurotransmitters stimulating adenylyl cyclase are expected to depolarize interneurons. Indeed application of serotonin has been shown to facilitate tonic activity in cat IN^27^. Based on the lack of an effect of SQ22536 we suppose that neuromodulators inhibiting adenylyl cyclase will not influence the membrane potential of IN. In fact adenosine had no detectable effect on the spontaneous firing or V_rest_ of cat IN^27^. It has been further suggested that activity of the cholinergic ascending brainstem exerts the strongest influence on IN^27^. Therefore the activation of *I*_h_ by PIP_2_ opens up the possibility for inhibition of HCN channels in IN via muscarinic ACh receptors coupled to Gq protein. Therefore the hyperpolarization of IN following ACh application seems to be based on the activation of a K^+^ leak current^27^ and the inhibition of *I*_h_. Thus the properties of *I*_h_ closely reflect the modulatory options of neurotransmitters in IN.

## Methods

### Experimental animals and slice preparation

All animal work has been approved by local authorities (review board institution: Landesamt für Natur, Umwelt und Verbraucherschutz Nordrhein-Westfalen; approval ID: 8.87–51.05.2010.117, 8.87–51.04.2010.A322). Experiments were performed on GAD67-EGFP^10^,^11^ mice ranging in age between P18-28 in accordance with the approved guidelines. Mice were sacrificed in isoflurane anesthesia and after surgically removing a skull cap caudal to the bregma, a block of brain tissue containing the thalamus was submerged in ice-cold aerated (O_2_) saline containing (in mM): sucrose, 200; PIPES, 20; KCl, 2.5; NaH_2_PO_4_, 1.25; MgSO_4_, 10; CaCl_2_, 0.5; dextrose, 10; pH 7.35, with NaOH. Thalamic slices (240–300 μm thickness) were prepared as coronal sections on a vibratome. Slices were transferred to a holding chamber and kept submerged (at 30 °C for 30 min) in artificial cerebrospinal fluid (ACSF) containing (in mM): NaCl, 125; KCl, 2.5; NaH_2_PO_4_, 1.25; NaHCO_3_, 24; MgSO_4_, 2; CaCl_2_, 2; dextrose, 10; pH adjusted to 7.35 by bubbling with carbogen (95% O_2_ and 5% CO_2_).

### Whole-cell patch clamp

*In vitro* recordings were performed on IN soma of the dLGN in a solution containing (in mM): NaCl, 120; KCl, 2.5; NaH_2_PO_4_, 1.25; HEPES, 30; MgSO_4_, 2; CaCl_2_, 2; dextrose, 10; pH 7.25 was adjusted with HCl. Voltage clamp recordings were performed in the presence of BaCl_2_ (0.5 mM) at a controlled room temperature (22 ± 1 °C). Current clamp recordings were performed at 33 ± 2 °C (In-Line Solution Heater, Harvard Apparatus, March, Germany). Individual cells were visually identified by infrared differential interference contrast video-microscopy (DIC-IR) and fluorescent imaging. Membrane currents were measured with glass microelectrodes (GC150T-10; Clark Electromedical Instruments, Pangbourne, UK) and filled with (in mM): K-gluconate, 95; K_3_-citrate, 20; NaCl, 10; HEPES, 10; MgCl_2_, 1; CaCl_2_, 0.5; BAPTA, 3; Mg-ATP, 3; Na_2_-GTP, 0.5. The internal solution was set to a pH of 7.25 with KOH and an osmolality of 295 mOsm/kg. A 0.2 μm pore size sterile filter (SUN-Sri, Rockwood, USA) was placed between the needle and the syringe to fill the patch electrodes. The free Ca^2+^ concentration of the internal solution was 45 nM (http://maxchelator.stanford.edu). The chlorinated silver recording electrode was connected to an EPC-10 amplifier (HEKA Elektronik, Lamprecht, Germany). Electrode resistances were in the range of 3-5 MΩ, with access resistances 6-14 MΩ. Series resistance compensation of >30% was routinely applied. Care was exercised to monitor series resistance and recordings were terminated whenever a significant increase (>20%) occurred. Voltage-clamp experiments were controlled by the software Pulse or PatchMaster (HEKA Elektronik) operating on an IBM-compatible personal computer. All recordings were corrected offline for a liquid junction potential of 10 mV (V_M_ = V_P_–10 mV; with V_M_ = membrane potential and V_P_ = pipette voltage).

The voltage protocol used to analyze *I*_h_^34^ was designed in order to increase the stability of whole-cell recordings (see Fig. 5B, inset). The pulse length was shortened by 500 ms with increasing hyperpolarization (1.5 s pulse length at −130 mV). In some recordings longer voltage steps were used (3.5 s pulse length at −130 mV). Steady-state activation of *I*_h_, p(V), was estimated by normalizing the mean tail current amplitudes (I) 100 ms (see Fig. 5B, arrow) after stepping to a constant potential from a variable amplitude step using equation 1:


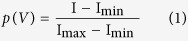


with I_max_ being the tail current amplitude for the voltage step from −130 mV to −100 mV and I_min_ for the voltage step from −40 mV to −100 mV, respectively. *I*_h_ activation was well accounted for by a Boltzmann function (equation 2) of the following form:


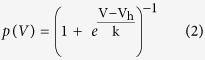


where V_h_ is the voltage of half-maximal activation and k the slope factor.

The current density was calculated by dividing the *I*_h_ amplitude at −130 mV by the membrane capacitance.

The time course of *I*_h_ activation in thalamic neurons at room temperature was approximated by a single-exponential function (equation 3)^14^:


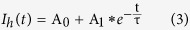


where *I*_h_ (*t*) was the current amplitude at time *t*, and *A*_0_, *A*_1_ and τ are axis intercept, amplitude coefficient, and time constant, respectively. Currents evoked by voltage steps to −130 mV were analyzed.

The reversal potential of *I*_h_ (E_h_) was determined by stepping for 2.5 s to a constant potential (−115 mV) followed by steps to varying potentials (−110 to −30 mV) and plotting the fully activated current vs. voltage (*I–V*) relationship of the tail current amplitude at 100 ms (see arrow in Fig. S1A)^32^. Linear fits were employed to the data points.

### Determination of the intrinsic electrophysiological properties of IN

Current clamp recordings were used to observe passive and active membrane properties. Analysis was performed according to established procedures^54^. Only cells with overshooting APs were included for analysis. Electrophysiological parameters were measured from responses to step current injections of 800 ms duration applied from the RMP. Injected currents varied between −120 to 130 pA (increments of 50 pA). In some experiments depolarizing current steps of 5 pA increments were used. Membrane input resistance (R_in_) was deduced from the slope of the current-voltage *I-V* relationship obtained from the current injections to −20 and 30 pA. Membrane time constants (τ_m_) were obtained by fitting single or double exponentials (FitMaster, HEKA Elektronik) to negative voltage deflections induced by hyperpolarizing current injections of −20 pA. The membrane capacitance (C_m_) was calculated using the equation: C_m_ = τ_m_/R_in_. For voltage clamp recordings C_m_ values were directly obtained from amplifier readings. The *I*_h_-dependent anomalous rectification or voltage sag (V_sag_) of the membrane potential was measured for potentials reaching a maximal negative value of −93.7 ± 4.3 mV (mean ± standard deviation; n = 228) and calculated as the relative change between the maximal (V_max_, typically reached within 200 ms) and steady state voltage deflection (V_ss_, at the end of hyperpolarizing current injection) using equation 4^55^:


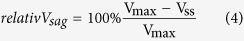


AP were detected by manually setting an amplitude threshold (V_thresh_; typically around −35 mV) and properties were determined for the first AP evoked by a depolarizing current step (+80 pA) using the FitMaster algorism (HEKA Elektronik; see supplementary information for details).

### Cluster analysis

A bias-free classification of EGFP-expressing neurons was based on an unsupervised cluster analysis^23^,^54^,^56^,^57^ using 12 electrophysiological parameters from 228 neurons recorded in dLGN under current clamp conditions. In order to achieve optimal clustering results, parameters were used which were variant in all observed cells but revealed no excessive variability from trial to trial. Based on these criteria, electrophysiological parameters used for cluster analysis were: C_m_, RMP, R_in_, τ_m_, relative V_sag_, V_thresh_, MaxY, fAHP (termed MinY by the analyzing software), MAXdt, MINdt, Dur, Integr (see supplementary information for details). Clustering was implemented using MATLAB (The MathWorks GmbH, Ismaning, Germany) and its statistical toolbox. The Thorndike procedure, where jumps in distance within clusters indicate prominent differences between groups, was used to examine resulting clusters^58^. By using this procedure IN were grouped in two clusters in a way that minimized the Euclidean distances between cells and cluster-centroids in a multi-parametric space. While Ward’s method has the advantage that the algorithm requires no definition of the number of clusters prior to analysis, the iterative nature of the process does not allow correction of miss-assigned cells during clustering. Therefore the K-means algorithm was used to eliminate potential errors of the Ward´s clustering process^23^,^24^,^25^. This method was initiated with setting K, the desired number of cluster centers. The algorithm assigned each observation to one of K corresponding clusters by minimizing the distance of the observation to the centers and moving observations between clusters until the sum of distances cannot be decreased further and K non-overlapping optimal groups were found. In the following cluster centers were chosen so as to correspond to the centroids of the clusters generated with Ward’s methods. The ideal number of clusters was subsequently defined by the K-value which indicated the highest mean silhouette value. In silhouette analysis, the value S(i) was computed for each data point by using equation 5:


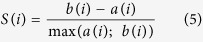


where for each data point i, a(i) and b(i) corresponded to the average distance between i and the points belonging to the same and the points of the closest cluster, respectively. A positive silhouette value indicated that on average, the cell is closer to the neurons of its own group than to neurons belonging to other clusters in the parameter space. A negative value indicated a potential misclassification. Thus, the mean silhouette value will be maximal for the optimal number of cluster number and decrease for higher or lower numbers of clusters thereby indicating a lower quality of clustering. In order to achieve maximal consistency of the clustering results, parameters independent from each other were used for K-means correction, namely C_m_, RMP, relative V_sag_, V_thresh_, MINdt, Dur, and Integr.

### Drugs

During experiments with 8-bromo-cyclic adenosine monophosphate (8-bromo-cAMP; water soluble; Sigma, Taufkirchen, Germany), 8-bromo-cyclic guanosine monophosphate (8-bromo-cGMP, Sigma) or phosphatidylinositol 4, 5-bisphosphate (PIP_2_, Echelon Biosciences Inc., Salt Lake City, USA) added to the recording pipette, properties of *I*_h_ were determined 10–15 min after obtaining the whole-cell configuration. ZD7288 (Tocris Bio-Techne, Wiesbaden, Germany) and EC18^36^ were directly dissolved in the external recording solution.

### Acute isolation of dLGN neurons

The dLGN was visually identified in coronal thalamic slices (400 μm) from mice (P16–P21), dissected, and transferred to a spinner flask with cold and oxygenated dissociation solution containing (mM): NaCl, 120; KCl, 5; MgCl_2_, 3; CaCl_2_, 1; PIPES, 20; glucose, 25; pH, 7.35. The solution was warmed up to 30 °C and then trypsin (1 mg/ml) was added. After several times of washing with the dissociation solution and triturating with fire-polished Pasteur-pipettes acutely isolated neurons were obtained. IN and TC neurons were visually identified by using morphological parameters^8^,^31^. Additionally green fluorescence was a definite characteristic of IN. Groups of 10 identified small IN (cell diameter <12 μm), large IN (cell diameter >12 μm) or TC neurons were collected in a sterile pipette, transferred into a PCR reaction tube and stored in liquid nitrogen.

### Multiplex and nested PCR

Random hexamer polynucleotides and the Sensiscript^TM^ reverse transcription kit (Qiagen, Hilden, Germany) were used for cDNA synthesis at 37 °C for 60 min. The efficiency of cDNA synthesis was controlled by PCR amplification of GAPDH. Following reverse transcription, the cDNAs of HCN1-HCN4 were amplified simultaneously using 2 μl of the reaction mixture. Nested PCR was performed in 40 μl reaction mixture containing: 0.125 U recombinant Taq polymerase (Invitrogen, Darmstadt, Germany), 2.5 mM MgCl_2_, 0.2 mM of each dNTP and 50 pmol of each primer. The cycling protocol was: 3 min at 94 °C; 45 cycles: 0.5 min at 94 °C, 0.5 min at 51 °C, 1 min at 72 °C; 7 min at 72 °C. Nested amplification was carried out individually for each target in 20 μl reaction mixtures using 2.5 μl from the first amplification product, 10 pmole of corresponding primers and 2.5 U Platinum Taq polymerase (Invitrogen). The cycling protocol was: 3 min at 94° C; 40 cycles: 30 sec at 94 °C, 0.5 min at 54 °C, 1 min at 72 °C; 7 min at 72 °C (see supplementary information for primer sequences).

### Immunofluorescence staining

Staining was performed as described earlier^59^. In brief, GAD67-EGFP mice were deeply anaesthetized with sodium pentobarbital (100 mg/kg) and transcardially perfused with ice-cold sodium phosphate-buffered saline (PBS; pH 7.4, 20 ml) followed by of 4% paraformaldehyde in PBS (50 ml). After removal, brains were post fixed for 2 h, and saturated overnight with 30% sucrose in PBS. Coronal slices (30 μm thickness) were cut on a Leica Frigomobil freezing microtome stage (Leica Microsystems GmbH, Wetzlar, Germany). Free-floating sections were permeabilized at room temperature and unspecific binding sites were blocked 2 h in PBS containing: 3% BSA, 0.3% Triton-X100 and 10% normal goat serum or horse serum. Primary antibodies (single staining: rabbit anti-nNOS, N7280, Sigma, 1:250 dilution; double staining: goat anti-nNOS, ab1376, Abcam, Cambridge, UK, 1:250 dilution in combination with rabbit anti-HCN2, APC-030, Alomone Labs, Jerusalem, Israel, 1:50 dilution) were dissolved in blocking solution. Slices were incubated overnight at 4 °C, washed three times (20 min each) in PBS, and thereafter incubated for 2 h at room temperature with the secondary antibodies (goat anti-rabbit Alexa Flour 568-conjugate; goat anti-rabbit Alexa Fluor-405-conjugate in combination with donkey anti-goat Alexa Fluor-594-conugate; Thermofisher, Schwerte, Germany; diluted 1:500 in blocking solution). After washing, slices were mounted on glass slides and preserved by ImmuMount (Thermofisher). Cells in the dLGN were analyzed using a laser scanning confocal microscope (NikoneC1plus, Nikon GmbH, Düsseldorf, Germany) equipped with a CFI75LWD16 × /0.8 NA objective (Nikon). To detect the fluorescence of EGFP, Alexa Fluor-568/594 and Alexa Fluor-405-conjugate the 488 nm line of an argon laser, a 543 nm and a 405 nm HeNe laser in combination with adequate emission filters (515 nm/30 nm, 605 nm/75 nm, and 450 nm/30 nm) were used, respectively.

### Determination of cell diameter in brain slices

For randomly chosen green fluorescent IN the largest cell diameter was determined manually in the confocal plane revealing the maximal soma shape by using EZ-C1 3.70 free viewer software (Nikon).

### Statistics

If not mentioned otherwise all results are presented as mean ± SEM. The number of experimental repetitions (n) reflects the number of cells (if not stated otherwise). For all experiments cells from at least 3 different slices from at least 3 different mice were used. By default statistical significance was tested using the nonparametric Mann-Whitney test (Graph Pad Prism software; Graph Pad, San Diego, CA; OriginLab software, Additive GmbH, Friedrichsdorf, Germany). For normally distributed data Student’s t-test was used. The Shapiro-Wilk test was used to identify normal distribution. For multiple comparisons one way or factorial ANOVA with Tukey’s or Bonferroni’s Post-hoc test was used. Differences were considered statistically significant if P < 0.05.

In order to assess the bimodal distribution of our data sets a Gauss function of the following form was used (equation 6):


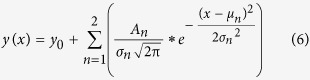


where y_0_ was the offset, A_n_ were areas under the curve, μ_n_ were centers of the distribution, and σ_n_ the widths of the distribution.

## Additional Information

**How to cite this article**: Leist, M. *et al*. Two types of interneurons in the mouse lateral geniculate nucleus are characterized by different h-current density. *Sci. Rep.* **6**, 24904; doi: 10.1038/srep24904 (2016).

## Supplementary Material

Supplementary Information

## Figures and Tables

**Table 1:** Table corresponding to Ward’s clustering (cf. Fig. 2A) and a clustering output generated by the K-means algorithm with the same sample (reassigned components are marked by (*)).

	Small	Large		
Small	88	15^*^	103	Ward’s clustering
Large	15^*^	110	125	
	103	125	228	
	K-means clustering			

Note that the clusters obtained by the two methods are mostly overlapping.
