# Supplementary material for: Two types of interneurons in the mouse lateral geniculate nucleus are characterized by different h-current density
Source: Sci Rep. 2016 Apr 28;6:24904. doi: 10.1038/srep24904 (PMC4848471; doi:10.1038/srep24904)
Supplement: Supplementary Information [file srep24904-s1.pdf]

Supplementary Information

Two types of interneurons in the mouse lateral geniculate nucleus are characterized by different h-current density

Michael Leist[1,#], Maia Datunashvilli[1,2,#], Tatyana Kanyshkova[1], Mehrnoush Zobeiri[1], Ania Aissaoui[1], Manuela Cerina[3], Maria Novella Romanelli[4], Hans-Christian Pape[1], Thomas Budde*[1]

[1]Institut für Physiologie I, Westfälische Wilhelms-Universität, Robert-Koch-Str. 27a, 48149 Münster, Germany.

[2]Laboratory of Sleep-Wakefulness Cycle Studies, Faculty of Arts and Science, Ilia State University, Kakutsa Cholokashvili Ave 3/5, Tbilisi 0162, Georgia.

[3]Institut für Physiologie I – Neuropathophysiologie, Albert-Schweitzer Campus 1, 48149 Münster, Germany.

[4]Department of Neurosciences, Psychology, Drug Research and Child Health, University of Florence, Via Ugo Schiff 6, 50019 Sesto Fiorentino, Italy.

[#]Authors contributed equally

*Corresponding author

A) Supplementary Methods

1) Determination of AP parameters

The following parameters obtained from current clamp recordings were analyzed: maximum potential reached by the AP during the depolarizing phase (MaxY); minimum potential following a single AP during the repolarization phase (MinY), thus representing the value of the fast afterhyperpolarization (fAHP); duration of the AP determined at the threshold level (Dur); integral of the AP with the threshold level used as baseline (Integr); rise time of the AP determined between the threshold level and MaxY (MAXdt); duration from the first threshold

crossing to MinY (MINdt); depolarization rate of the AP calculated by the equation: (MaxY - V_{thresh}) / MAXdt; AP repolarization rate of the AP calculated by the equation: (MaxY - MinY) / MINdt (note that MinY has negative values). The firing frequency was deduced by calculating the dividing the number of AP induced by a depolarizing current step by the pulse length.

2) Sequences of primers used for multiplex and nested PCR

The following primers were used:

Multiplex primer

HCN1 (nucleotides985-1883); accession No. AJ225123:

for: 5'-TCTTGC GGTTATTAC GCC TT-3';

rev: 5'-TTT TCT TGC CTA TCC GAT CG-3'.

HCN2 (nucleotides810-1774) accession No.AJ225122:

for: 5'-TAC TTG CGT ACG TGG TTC GT-3';

rev:5'-GAAATAGGAGCCATC CGACA-3'.

HCN3 (nucleotides 1242-2322); accession No. AJ225124:

for: 5'-CGC ATC CAC GAG TAC TAC GA-3';

rev: 5'-CAC TTC CAG AGC CTT TAC GC-3'.

HCN4 (nucleotides 295-1314); accession No. AF064874:

for: 5'-TCT GAT CAT CAT ACC CGT GG-3';

rev:5'-GAA GAC CTC GAA ACG CAA CT-3'.

Nested primer

HCN1 (nucleotides 1612-1902, 290 bp); accession No. AJ225123:

for: 5’-CTC TTT TTG CTA ACG CCG AT-3’;

rev: 5’-CAT TGA AAT TGTCCACCGAA-3’.

HCN2 (nucleotides 1181-1550, 369 bp); accession No. AJ225122:

for: 5’-GTG GAG CGA GCT CTA CTC GT-3’;

rev: 5’-GTT CAC AAT CTC CTC ACG CA-3’.

HCN3 (nucleotides 1808-2040, 232 bp); accession No. AJ225124:

for: 5’-GCA GCA TTT GGT ACA ACA CG-3’;

rev: 5’-AGC GTC TAG CAG ATC GAG CT-3’.

HCN4 (nucleotides 1110-1278, 168 bp); accession No. AF064874:

for: 5’-GAC AGC GCA TCC ATG ACT AC-3’;

rev: 5’-ACA AAG TTG GGA TCTGCG TT-3’.

B) Supplementary Figures

Figure S1: Reversal of I_h in small and large IN in dLGN.

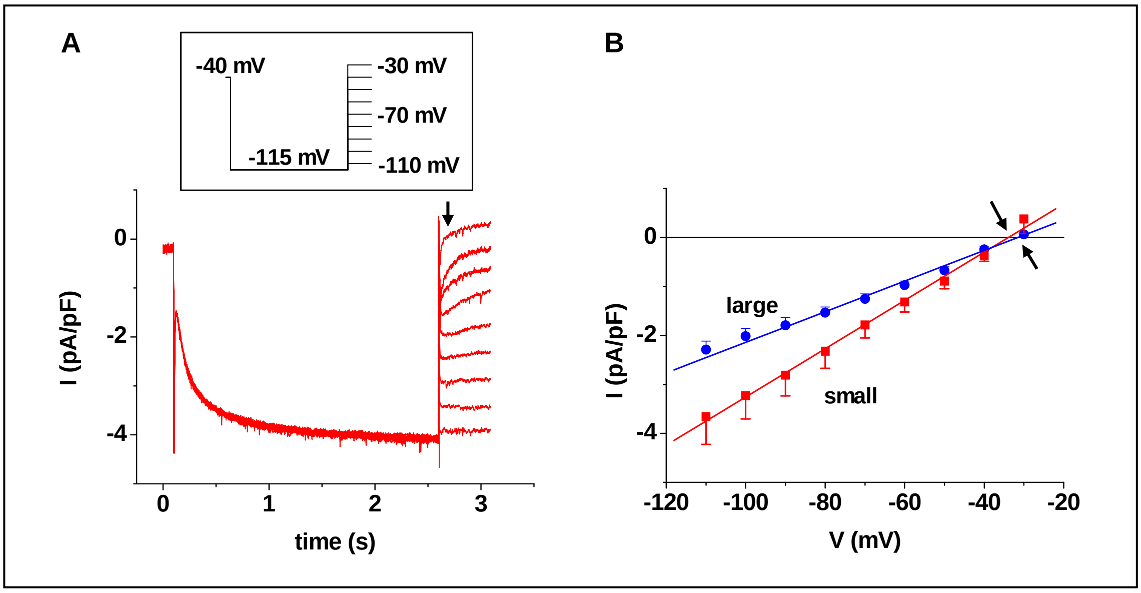

(**A**) Current traces evoked by a hyperpolarizing step to -115 mV from a holding potential of -40 mV (see inset) in a small IN. The post-step test potential was varied between -110 and -30 mV. Tail current amplitudes 100 ms after the onset of the test potential were measured (see arrow). (**B**) Plots of tail current amplitudes vs. test potential for small (red squares) and large (blue circles) IN. The solid lines represent the best fitting straight lines with zero current a potential of -33 mV and -31 mV for small and large IN, respectively (see arrows).

Figure S2: Properties of I_h in dLGN TC neurons of EGFP-expressing mice.

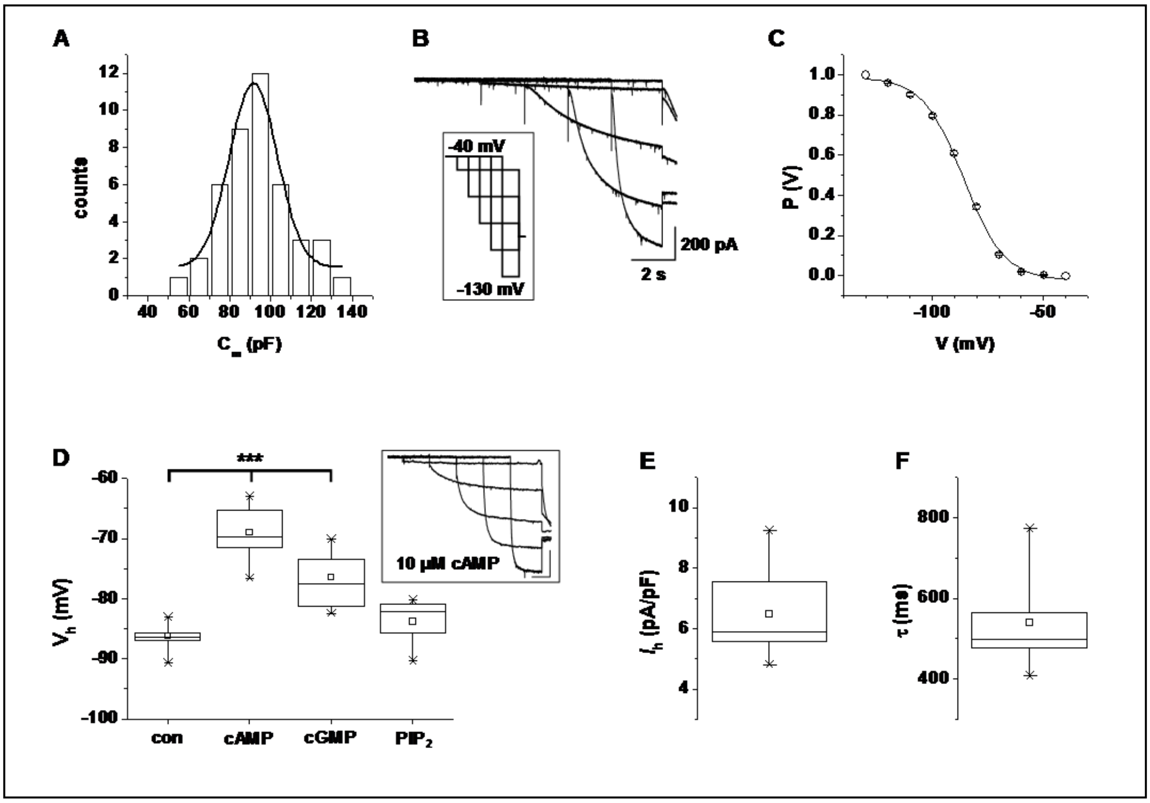

(**A**) Frequency distribution of C_m of TC neurons recorded under voltage clamp conditions. The ordinate represents the number of cells within each 10 pF class along the abscissa. Note the unimodal C_m distribution for the total TC neuron population in dLGN. (**B**) Representative I_h recording from a TC neuron in dLGN is shown. The inset shows the voltage clamp protocol. (**C**) The mean steady-state activation curve from TC neurons is shown. The solid line represents the best approximation of a Boltzmann function to the data points. (**D**) Box plots of V_h values under control conditions (con) with cAMP (10 µM), cGMP (10 µM) or PIP_2 (10 µM) included to the pipette solution of independent populations of TC neurons are shown (one way ANOVA: $F = 55.93$, $p < 0.001$; Tukey's posthoc: con vs. cAMP, $p < 0.001$; con vs. cGMP, $p < 0.001$). The inset shows representative current traces from a TC neuron in the presence of 10 µM intracellular cAMP. Scale bars represent 2s and 200 pA. (**E, F**) Box plots of I_h current density determined at -130 mV (**E**) and time constants of activation (**F**) in TC neurons are shown.

C) Supplementary Tables

Table S1. Electrophysiological parameters (mean ± SEM) of dLGN IN recorded from GAD67-EGFP mice.

Parameter	Small	Large	t-test P-value
number of cells	125	103	
$V_{sag} = V_{max} - V_{ss}$ [mV]	8.12 ± 0.27	4.23 ± 0.27	P < 0.001 ***
relative V_{sag} [%]	8.56 ± 0.29	4.56 ± 0.29	P < 0.001 ***
R_{in} [GΩ]	0.40 ± 0.01	0.33 ± 0.02	P = 0.001 **
τ_m [ms]	26.96 ± 1.04	43.76 ± 2.20	P < 0.001 ***
C_m [pF]	69.52 ± 1.94	132.73 ± 3.17	P < 0.001 ***
V_{sag}/C_m [µV/pF]	130.57 ± 6.88	33.31 ± 2.02	P < 0.001 ***
RMP [mV]	-62.36 ± 0.57	-64.88 ± 0.66	P = 0.004 **
V_{thresh} [mV]	-35.37 ± 0.45	-35.41 ± 0.45	n. s.
Dur [ms]	1.91 ± 0.08	2.20 ± 0.07	P = 0.008 **
Integr [µVs]	30.14 ± 1.17	35.82 ± 1.39	P = 0.002 **
MaxY [mV]	19.55 ± 1.19	19.28 ± 1.19	n. s.
MAXdt [ms]	1.10 ± 0.06	1.33 ± 0.06	P = 0.008 **
Depolarization rate [V/s]	68.74 ± 3.98	53.73 ± 3.26	P = 0.005 **
MinY [mV]	-45.16 ± 0.47	-43.05 ± 0.56	P = 0.004 **
MINdt [ms]	3.20 ± 0.10	3.83 ± 0.12	P < 0.001 ***
Repolarization rate [V/s]	22.52 ± 0.85	18.06 ± 0.69	P < 0.001 ***
Firing rate @ 30 pA [Hz]	4.19 ± 0.68	0.40 ± 0.15	P < 0.001 ***
Firing rate @ 80 pA [Hz]	10.4 1 ± 1.05	2.02 ± 0.35	P < 0.001 ***
Firing rate @ 130 pA [Hz]	11.08 ± 1.19	3.20 ± 0.43	P < 0.001 ***

The assignment to the groups of small and large IN was done based on k-mean correction using the underlined set of parameters (C_m, RMP, relative V_{sag}, V_{thresh}, MINdt, Dur, Integr).
